# The contribution of neuropilin‐1 in the stability of CD4^+^CD25^+^ regulatory T cells through the TGF‐β1/Smads signaling pathway in the presence of lipopolysaccharides

**DOI:** 10.1002/iid3.551

**Published:** 2021-11-10

**Authors:** Yulei Gao, Xiang Zhang, Ziyi Wang, Yuting Qiu, Yancun Liu, Songtao Shou, Yanfen Chai

**Affiliations:** ^1^ Department of Emergency Medicine Tianjin Medical University General Hospital Tianjin PR China; ^2^ Department of Emergency Medicine Rizhao People's Hospital of Shandong Province Rizhao PR China

**Keywords:** lipopolysaccharides, negative immunoregulation, neuropilin‐1, regulatory T cells, Sepsis, transforming growth factor‐β1

## Abstract

**Introduction:**

This study investigates the synergistic effect of TGF‐β1 and Nrp‐1 on CD4^+^CD25^+^ T_regs_' stabilization, and the associated pathways of signal transduction, in vitro models in the presence of LPS.

**Materials and Methods:**

Spleen CD4^+^CD25^+^ T_regs_ cells of mice models in the presence of LPS, were transfected with an shRNA targeting Nrp‐1, Smad2, or Smad3, may or may not be treated with recombinant TGF‐β1. Followed by subsequent determination of cellular proliferation, rate of apoptosis, observation of the Foxp3, CTLA‐4, and TGF‐β1^m+^ expression levels, foxp3‐TSDR methylation, secretion levels of the inhibitory cytokines IL‐10 and TGF‐β1, and Smad2/3 of CD4^+^CD25^+^ T_regs_ expression.

**Results:**

A remarkable stimulation in CD4^+^CD25^+^ T_regs_' stability is noted after administering recombinant TGF‐β1 in the presence of LPS, and promoted cellular viability, increased Foxp3, CTLA‐4, and TGF‐β1^m+^ expression, and elevated secretion of IL‐10 and TGF‐β1. This also inhibited the apoptosis and methylation of foxp3‐ TSDR of CD4^+^CD25^+^ T_regs_. The shRNA transfection silenced Nrp‐1 and Smad3, but not Smad2, resulting in the suppression of the recombinant TGF‐β1‐mediated effects in the presence of LPS.

**Conclusions:**

According to the results, Nrp‐1 mediates TGF‐β1 to improve the stability of regulatory CD4^+^CD25^+^ T cells and maybe a possible therapeutic target with the ability to improve the CD4^+^CD25^+^ T_regs_ associated negative immunoregulation that is related to the TGF‐β1/Smads cell signaling during sepsis.

## Graphical abstract

Our results suggest that Nrp‐1 is beneficial for TGF‐β1 to enhance the stability of CD4^+^CD25^+^ T_regs_, and may represent a novel therapeutic target with the potential to improve the CD4^+^CD25^+^ T_regs_‐related primary negative immunoregulation associated with the TGF‐β1/Smads signaling pathway in sepsis.

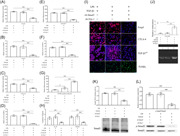

## 1 INTRODUCTION

The dysregulation in a host that causes life‐threatening organ dysfunction in response to an infection is called sepsis.^1^, ^2^, ^3^ Strong experimental and clinical evidence shows that sepsis is a complicated pathogenic disorder that needs to be further studied for a better understanding.^1^ Long‐term immunosuppression is noted during the initial stages of sepsis both in animal models and sepsis patients, which is associated with the loss of B or T lymphocytes, epithelial cells of the gut, dendritic cells, and thymocytes. The loss of these immune cells can be defined as immune paralysis.^2^ Patients still show prominent symptoms of immune dysfunction post‐recovery, such as cellular immunosuppression, and susceptibility to secondary infections.^2^, ^4^ To counter this condition, it is important to further investigate the underlying immunosuppression mechanisms and the development of new measures for the regulation of immune response during sepsis.^2^, ^3^, ^4^

Regulatory T cells (T_regs_) are an immunosuppressive subpopulation of CD4^+^ T lymphocytes characterized by expression of forkhead box protein P3 (Foxp3) transcription factor, and majorly involved in maintaining immune homeostasis and recognition of self‐cells by the body's immune system.^5^ The proportion of peripheral T_regs_ increases during the development of sepsis and septic shock, which is associated with sepsis‐induced immunosuppression and multiple organ dysfunction syndromes (MODS) via both TGF‐β1/Smads‐dependent and ‐independent pathways.^6^, ^7^, ^8^ Sepsis is a serious complication caused by deregulated inflammatory response against severe infections induced by endotoxin/lipopolysaccharide (LPS). In particular, infections caused by Gram‐negative pathogens mediates the release of LPS and activates the signaling of Toll‐like receptors‐4 signaling during sepsis. Based on our study before, during sepsis, the overexpression of TLR‐4 on the surface of Tregs is observed. This indicated that it affects the processes of immunopathology and immune microenvironment by altering certain signaling pathways or cytokine networks.^2^, ^4^, ^5^, ^7^

Neuropilin‐1 (Nrp‐1) is primarily regarded as a receptor for both semaphorins (Sema, such as Sema3A) and vascular endothelial growth factor (VEGF) family.^9^ It also acts as a marker of T_regs_, which often also express Nrp‐1 and TGF‐β1.^10^, ^11^ Studies have demonstrated that Nrp‐1 functions as a membrane‐associated TGF‐β1 (TGF‐β1^m+^) coreceptor, augmenting canonical Smad2/3 signaling, while loss of Nrp‐1 leads to reduced Smad2/3 phosphorylation.^12^, ^13^ However, in a few studies researchers have examined the role and associated mechanisms of Nrp‐1 in immunosuppression and whether it affects the cell viability of CD4^+^CD25^+^ T_regs_ in sepsis. Previously, Tuftsin, an Nrp‐1 ligand, was shown to inhibit the negative immunoregulatory effects of Nrp‐1^high^ CD4^+^CD25^+^ T_regs_ and improved the survival outcomes of septic mice.^14^ Moreover, Nrp‐1^high^CD4^+^CD25^+^ T_regs_ revealed primary negative immunoregulatory activity in reaction to sepsis, while administration of a recombinant version of Nrp‐1 polyclonal antibody markedly lowered the demethylation of Foxp3‐TSDR in a dose‐dependent manner under LPS stimulation.^15^

In the present study, we transfected CD4^+^CD25^+^ T_regs_ with a short hairpin (sh) RNA targeting Nrp‐1 (sh‐Nrp‐1), Smad2 (sh‐Smad2) or Smad3 (sh‐Smad3), and analyzed the changes in Smads‐related protein expression, along with CD4^+^CD25^+^ T_regs_ stability levels, to study Nrp‐1 involvement in the TGF‐β1/Smads‐associated signal transduction pathway.

## 2 MATERIALS AND METHODS

### 2.1 Mice and ethics statement

6 to 8 weeks old, inbred male mice (~20 g; C57BL/6J) were groomed in standard care at the Animal Center of the Chinese Academy of Medical Sciences Laboratory, No. SCXK‐Jing‐2014‐0004, Beijing, China. The mice were kept in a 12 h dark/light cycle at 25°C and in an atmosphere of 60% with an adequate diet supply, following the animal care guidelines of the Tianjin Medical University General Hospital. All experiments were performed per the rules of the National Institute of Health Guidelines for the Care and Use of Laboratory Animals (Guide for the Care and Use of Laboratory Animals. 8th ed., National Academies Press (US), 2011.) after the approval of the review board of scientific investigation at Tianjin Medical University General Hospital (Approval no. ZYY‐DWFL‐IRB‐001F‐01).

### 2.2 Collection of CD4^+^CD25^+^ T_regs_ from spleen

Single‐cell splenic suspensions were produced after collecting spleens from the mice followed by density gradient centrifugation supplied by Ficoll‐Paque (Nanjing Keygen Biotech). Single‐cell splenic suspensions were used to collect CD4^+^CD25^+^ T_regs_ and CD4^+^CD25^−^ T cells by CD4^+^CD25^+^ T_regs_ isolation kits that contain a mix of 1‐ml monoclonal biotin‐conjugated antibodies to neutralize the CD8a, CD45R, CD11b, CD49b, and Ter‐119 cells of the mice under study, 2 ml microbeads to neutralize biotin, 1 ml of mouse CD25 antibodies conjugated with phycoeryanate, and 1 ml of anti‐ phycoeryanate microbeads supplied by Miltenyi Biotec GmbH) and a separator supplied by MiniMACS^tm^ (Miltenyi Biotec GmbH) as per the instructions of the manufacturer.

### 2.3 Experimental design

CD4^+^CD25^+^ T_regs_ were subsequently seeded on 96‐well cell culture plates at a cell concentration of 2 × 10^5^/well, and treated with anti‐CD3 (5 μg/ml) and anti‐CD28 (2 μg/ml) antibody for polyclonal activation of T cells, respectively. Cells were then transfected with sh‐Nrp‐1, sh‐Smad2, or sh‐Smad3 via lentivirus transduction, with or without administration of recombinant TGF‐β1, and in the presence of LPS (100 ng/ml, *Escherichia coli* 0111:B4, Sigma‐Aldrich) to simulate the environment of sepsis. After being stimulated, the viability and stability, as well as TGF‐β1/Smads signaling pathway of CD4^+^CD25^+^ T_regs_ were determined.

### 2.4 Making and transfection of short hairpin (sh) RNA targeted at Nrp‐1 and Smad2/3

The small interfering (si)RNA sequences and shRNA that were designed by Shanghai GenePharma Co., based on the gene sequence provided by GenBank. targeted Nrp‐1 (sh‐Nrp‐1:5′ AACCAGACACAGCTTCTTCCCAGTATATTCAAGAGATATACTGGGAAGAAGCTGTGATCTGTTTTTTC‐3′), Smad2 (sh‐Smad2: 5′‐GCCAGUUACUUAUUCAGAATT‐3′), and Smad3 (sh‐Smad3: 5′‐GCUGUUCCAGCGUGUCUUATT‐3′) and shRNA duplex (GenePharma Co) was considered a negative control. 293 T cells were used for the packaging of recombinant retroviruses according to BD Retro‐X^tm^ Universal Packaging System supplied by Clontech, Toyobo. The concentration of siRNA was 100 nmol/L, and the culture medium was replaced with a complete medium 6 h later. After 48 h, the supernatant was collected and diluted continuously. The total protein concentration was determined by bicinchoninic acid assay (BCA). Knockout (KO) experiments were carried out with the supernatant containing the optimal concentration of virus particles. A lentivirus transduction kit supplied by Vira Ductin^tm^ (Cell Biolabs, INC) was used to carry out transfection. Based on the determination of a valid siRNA target sequence, the corresponding DNA template strand and complementary strand encoding shRNA hairpin structure were designed and synthesized. The constructed recombinant vector plasmid was cotransfected with lentivirus packaging plasmid PRSV‐Rev, pMDLG/pRRE, and pvSV‐G, respectively, according to the mass ratio of 2:1:1:1. After 48 h, the supernatant was collected and centrifuged for 5 min at 3000 r/min at 4°C. The virus precipitate was resuspended at a concentration of 5 μmol/L and stored in a −80°C refrigerator. CD4^+^CD25^+^ T_regs_ were transfected with virus particles after multiple dilution (1:10, 1:20, and 1:200). transduction of CD4^+^CD25^+^ T_regs_ was carried out with sh‐Nrp‐1, sh‐Smad2, or sh‐Smad3 vectors as per the manufacturer's instructions. SYBR Green PCR mixed RT‐QPCR was used to detect the transfection efficiency of sh‐Nrp‐1, sh‐Smad2, or sh‐Smad3 in CD4^+^CD25^+^ T_regs_. RT‐qPCR amplification consisted of denaturation at 95°C for 1 min, followed by 40 cycles of 15 s at 95°C and 40 s at 60°C, and was done in a Sequence Detection System (Agilent Technologies). The interference efficiency of shRNA on Nrp‐1, Smad2, and Smad3 expression of CD4^+^CD25^+^ T_regs_ was above 50%, respectively.

### 2.5 3‐(4,5‐Dimethylthiazol‐2‐yl)‐2,5‐diphenyltetrazolium bromide (MTT) assay

The medium was refreshed and 10 ml of MTT reagent was added to each well to initiate the assays (Ameresco). The cells were then incubated for 4 h. Ultimately, the medium in each well was replaced with dimethyl sulfoxide (100 ml) supplied by Ameresco. The absorbance was read at a wavelength of 490 nm in a microplate photometer supplied by Spectra MR, Dynex.

### 2.6 Immunofluorescence analysis

For immunofluorescence analysis, the CD4^+^CD25^+^ T_regs_ were seeded on glass coverslips, washing was done using phosphate buffer saline, and 4% paraformaldehyde supplied by Nanjing Keygen Biotech, was used for fixation for 30 min followed by an incubation of 20 min with rabbit anti‐mouse Foxp3/CTLA‐4/Nrp‐1/TGF‐β1^m+^ antibodies supplied by Abcam, at 4°C. Washing was repeated and incubation of the cells was done at 4°C for 30 min with goat anti‐rabbit IgG conjugated with FITC/APC supplied by Jackson, Southern Biotechnology Associations, and Molecular Probes. A suspension of CD4^+^CD25^+^ T_regs_ and 1 ml permeabilization solution was made at 25°C and kept for 30 min and then the solution was seeded on glass coverslips followed by a washing step using phosphate‐buffered saline, then fixation of the suspension was done in 4% paraformaldehyde for 15 min at 25°C. Afterward, a 10% normal goat serum solution was used for 30 min at 25°C to block nonspecific antibody binding (Beijing Solarbio Science & Technology Co., Ltd.). Followed by an incubation period with rabbit anti‐mouse p‐Smad2 (1:100), Smad2 (1:100), p‐Smad3 (1:100), and Smad3 (1:100) antibodies (purchased from Abcam) for 12 h at 4°C. Washing was repeated followed by cellular incubation for 60 min at room temperature with goat anti‐rabbit IgG conjugated with FITC/APC (1:200, Southern Biotech). Fluorescence microscopy was performed for the analysis of immunofluorescence. Data collection and processing were done using ImageJ.

### 2.7 Terminal deoxynucleotidyl transferase (TdT) dUTP nick‐end labeling (TUNEL) assay

A single‐step apoptosis TUNEL Assay Kit was used (detection of fluorescein isothiocyanate [FITC]‐dUTP fluorescence; Solarbio) following the protocol set by the manufacturer. Immunofluorescence microscopy was performed for the detection of immunofluorescence (OLYMPUS). Green fluorescence of 525 nm wavelength was detected. Data collection and processing were done by ImageJ.

### 2.8 Enzyme‐linked immunosorbent assay

Supernatants were collected through enzyme‐linked immunosorbent assay kits to measure IL‐10 and TGF‐β1 levels (Excell Biol), following specified protocols by the manufacturer.

### 2.9 RT‐qPCR

The total RNA purification from 1 × 10^6^ cells/group was performed utilizing the NucleoSpin RNA II Kit supplied by Macherey‐Nagel following their protocols. The expression levels of mRNA of Foxp3/Ctla4/Tgfb1 were noted by running a qPCR reaction prepared in SYBR Green qPCR mix. The primers used for mouse gene Foxp3 were; F: 5′‐CAGCTGCCTACAGTGCCCCTAG‐3′, R: 5′‐CATTTGCCAGCAGTGGGTAG‐3′, primers used for mouse gene, Ctla4 were; F: 5′‐CGCAGATTTATGTCATTGATCC‐3′, R: 5′‐TTTTCACATAGACCCCTGTTGT‐3′ and for mouse gene, Tgfb1 primers used were; F: 5′‐AACAATTCCTGGCGTTACCTT‐3′, R: 5′‐GAATCGAAAGCCCTGTATTCC‐3′. PCR cycling conditions comprised of an initial denaturation step of 1 min at 95°C, the next step was to set up the reaction at 40 cycles of 15 s at 95°C and the final phase was set up for 40 s at 60°C. PCR reaction was performed in a Sequence Detection System (Agilent Technologies).

### 2.10 Methylation‐specific qPCR

Methylation‐specific q‐PCR was performed to determine the methylation pattern of foxp3‐TSDR as indicated.^15^ DNA was isolated from splenic CD4^+^CD25^+^ T_regs_ using the QIAamp DNA Mini‐kit (Qiagen) according to the manufacturer's guidelines, BisulFlash DNA Modification Kit (Epigentek) was used to modify DNA with sodium bisulfite. To deep amplicon analysis of the foxp3‐TSDR, we amplified bisulfite‐treated mouse DNA with tagged primers by using the AmpliTaq Polymerase Kit (Life Technologies) according to the manufacturer's guidelines. The PCR products were purified using the QIAEX II Gel Extraction Kit (Promega) according to the standard protocol. Two different labeled TaqMan probes (Promega) were designed to specifically bind the methylated or unmethylated of foxp3‐TSDR target sequences in mouse quantitative analysis of methylated alleles assays.

### 2.11 Electrophoretic mobility shift assay

Nuclear extraction was done for electrophoretic mobility shift assay (EMSA) after harvesting CD4^+^CD25^+^ T_regs_ (Thermo Fisher Scientific). These nuclear extracts were incubated with DNA oligonucleotide probes that were labeled with biotin at 3ʹ end, targeting the sequence at Smad2/3 binding sites. For the detection of Smad 2/3 activity, an anti‐Smad2/3 antibody was used that had DNA binding capability. To check the specificity of the probes, a mutated biotin‐free version of the probe was used. The mixture was incubated, followed by the analysis of the target as well as DNA samples on 5% polyacrylamide gel.

### 2.12 Western blot assay of p‐Smad2/Smad2 and p‐Smad3/Smad3

1× Nupage LDS lysis buffer supplied by Life Technologies, Carlsbad, CA, USA was used for the lysis of regulatory CD4^+^CD25^+^ T cells. The addition of phosphatase inhibitors supplied by Life Technologies was left as a choice. After lysis, cellular incubation was carried for 10 min at 95°C. The protein concentrations were quantified using the BCA (Life Technologies). A 10%–15% SDS‐PAGE was performed to extract equal protein quantities extracted from the lysed cells (Boster), followed by blotting onto nitrocellulose membranes. 3% BSA solution (Boster) was used to block the membranes for 2 h at 25°C, followed by incubation with primary antibodies (1:1000) at 4°C until the next morning. Rabbit antibodies including anti‐mouse anti‐Smad2, anti‐p‐Smad2, anti‐Smad3, and anti‐p‐Smad3 were supplied by Abcam. Afterward, horseradish peroxidase‐conjugated with goat anti‐rabbit IgG (Abcam) secondary antibodies were poured onto the membranes followed by an incubation of 1.5 h at room temperature. Lab Works^tm^ imaging and analysis system by UVP was used for data collection and processing.

### 2.13 Statistical analysis

One‐way ANOVA and Tukey's procedure using SPSS version 24 (IBM Corp.) was used to analyze the data (*n* = 6 per group) and noted as the mean ± standard deviation. To analyze the significance between the two groups, we used an unpaired Student's *t*‐test. Results were noted by considering *p < .05, **p < .01, ***p < .001 as a significant statistical difference.

## 3 RESULTS

### 3.1 Nrp‐1 and TGF‐β1 promoted the viability of CD4^+^CD25^+^ T_regs_ in the presence of LPS

**Figure 1:**
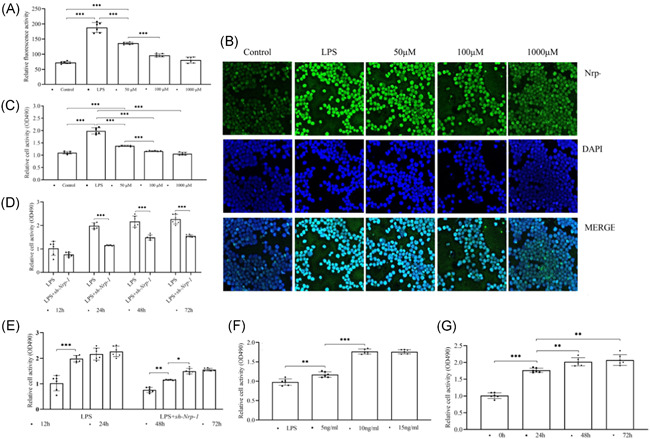
The effect of Nrp‐1 and TGF‐β1 on cell viability of CD4^+^CD25^+^ T_regs_ in sepsis induced by LPS. Expression of Nrp‐1 was decreased in CD4^+^CD25^+^ T_regs_ by transfection of sh‐Nrp‐1 through lentiviral infection in a concentration‐dependent manner (A, B). Sh‐Nrp‐1 transfection reduced the viability of CD4^+^CD25^+^ T_regs_ in a concentration (C)‐ and time (D, E)‐dependent manner. CD4^+^CD25^+^ T_regs_ were treated with recombinant TGF‐β1. Recombinant TGF‐β1 enhanced the viability of CD4^+^CD25^+^ T_regs_ in a dose (F)‐ and time (G)‐dependent manner

As opposed to the control group, Nrp‐1 expression (Figure 1A,B) and viability (Figure 1C) of CD4^+^CD25^+^ T_regs_ were profoundly elevated following LPS induction for 24 h (*p *< .001). Compared to the LPS group, Nrp‐1 expression (Figure 1A,B) and viability (Figure 1C) of CD4^+^CD25^+^ T_regs_ were significantly reduced following transfection with various concentrations of sh‐Nrp‐1 (50–1000 μM) and culture (*p* < .001) for 24 h, particularly at 100 μM concentration, and there was no difference between 100 and 1000 μM (*p* > .05). The viability of CD4^+^CD25^+^ T_regs_ was also lowered considerably in contrast with the LPS group after transfection with sh‐Nrp‐1 at the 100 μM concentration and 24, 48, or 72 h of culture (Figure 1D, *p* < .001), and especially when cultured for 48 h in comparison to 24 h group (Figure 1E, *p* < .05). When CD4^+^CD25^+^ T_regs_ were administered with different doses of recombinant TGF‐β1 and cultured for 24 h, their viability enhanced significantly at the dose of 10 ng/ml relative to that at 5 ng/ml (Figure 1F, *p* < .001), there was no difference between the 10 and 15 ng/ml doses (Figure 1F, *p *> .05). CD4^+^CD25^+^ T_regs_ were further cultured with recombinant TGF‐β1 at 10 ng/ml dose for 24, 48, and 72 , and the results indicated that the viability of CD4^+^CD25^+^ T_regs_ was significantly enhanced at 48 h when compared to their viability at 24 h (Figure 1G, *p* < .01).

### 3.2 Nrp‐1 silencing weakened the stimulated stability of CD4^+^CD25^+^ T_regs_ mediated by recombinant TGF‐β1 in the presence of LPS

**Figure 2:**
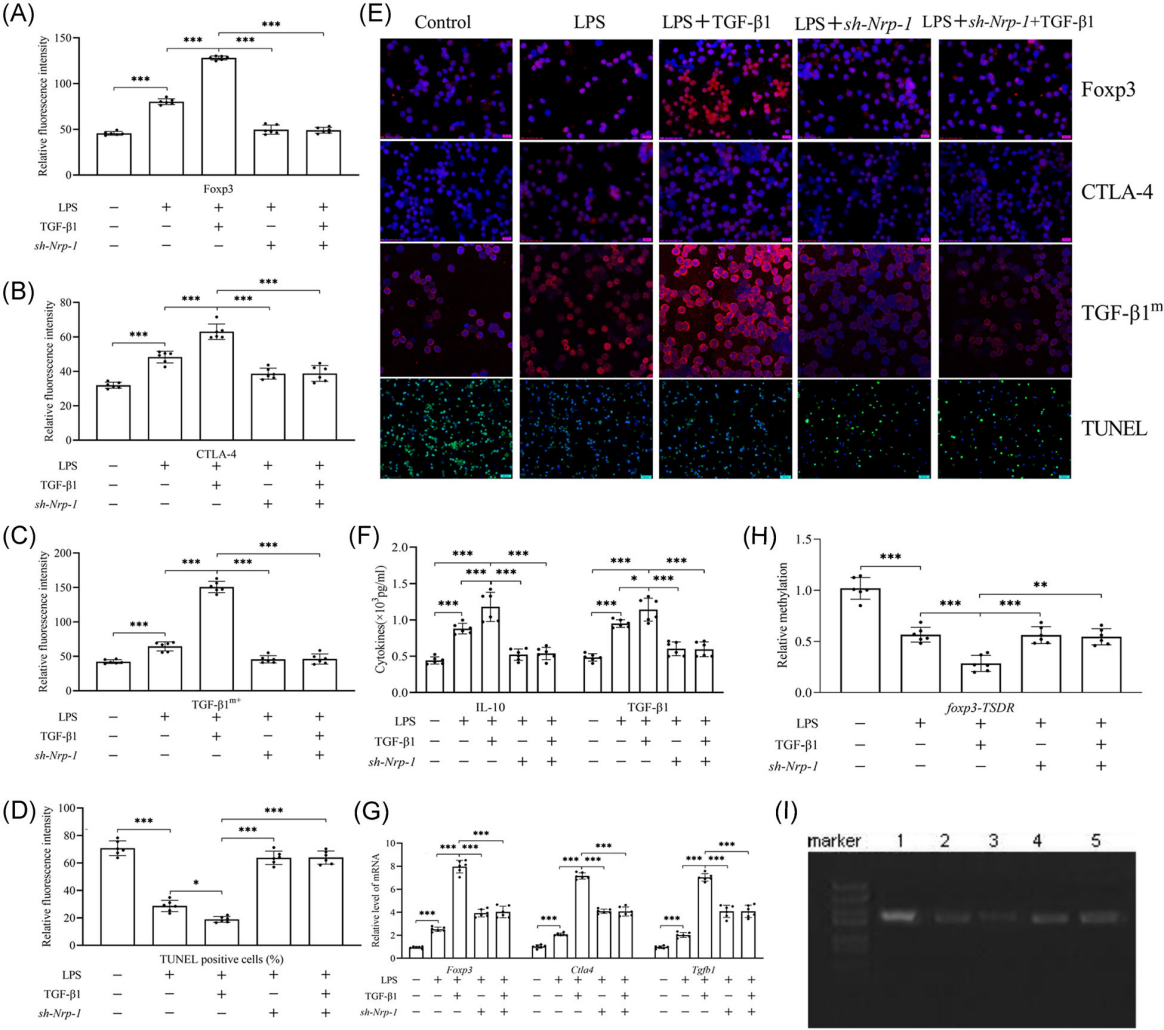
Nrp‐1 silencing effects on recombinant TGF‐β1‐mediated promotion of the CD4^+^CD25^+^ T_regs_ stability sepsis induced by LPS. sh‐Nrp‐1 transfection using infection by lentivirus weakened the stability of CD4^+^CD25^+^ T_regs_ after stimulation by LPS independent of recombinant TGF‐β1. Descriptive immunofluorescence images and statistical figures of the expression of Foxp3 (A, E), CTLA‐4 (B, E), and TGF‐β1^m+^ (C, E) at the level of protein, and CD4^+^CD25^+^ T_regs_ apoptosis level (D, E). The TGF‐β1 and IL‐10 secretion levels (F). The mRNA expression of ctla4, foxp3, and tgfb1 in regulatory CD4^+^CD25^+^ T cells (G). The methylation of foxp3‐TSDR (H, I)

After comparison with the control group, CD4^+^CD25^+^ T_regs_' stability was improved predominantly following LPS induction along with increased expression levels of Foxp3 (Figure 2A,E,G), CTLA‐4 (Figure 2B,E,G), and TGF‐β1^m+^ (Figure 2C,E,G) at the level of gene and protein (*p* < .001), elevated secretion levels of TGF‐β1 and IL‐10 (Figure 2F, *p* < .001), lowered apoptosis (Figure 2D,E, *p* < .001), and demethylated foxp3‐TSDR (Figure 2H,I, *p* < .001). After the recombinant TGF‐β1 (10 ng/ml) was administered it further stabilized the CD4^+^CD25^+^ T_regs_ (*p* < .05, 0.01, or 0.001) as compared to the LPS group while Nrp‐1 knockdown by transfection with 100 μM sh‐Nrp‐1 in CD4^+^CD25^+^ T_regs_ of the spleen resulted in a considerable decline in the stability of these cells (*p* < .05, 0.01, or 0.001). Moreover, compared with the only administration of recombinant TGF‐β1 group, Nrp‐1 silencing greatly reduced the stability of CD4^+^CD25^+^ T_regs_ that was induced by recombinant TGF‐β1 when sh‐Nrp‐1 were co‐administered (*p* < .01 or 0.001).

### 3.3 Weakened activity of the TGF‐β1/Smads signaling pathway of CD4^+^CD25^+^ T_regs_ due to Nrp‐1 silencing in the presence of LPS

**Figure 3:**
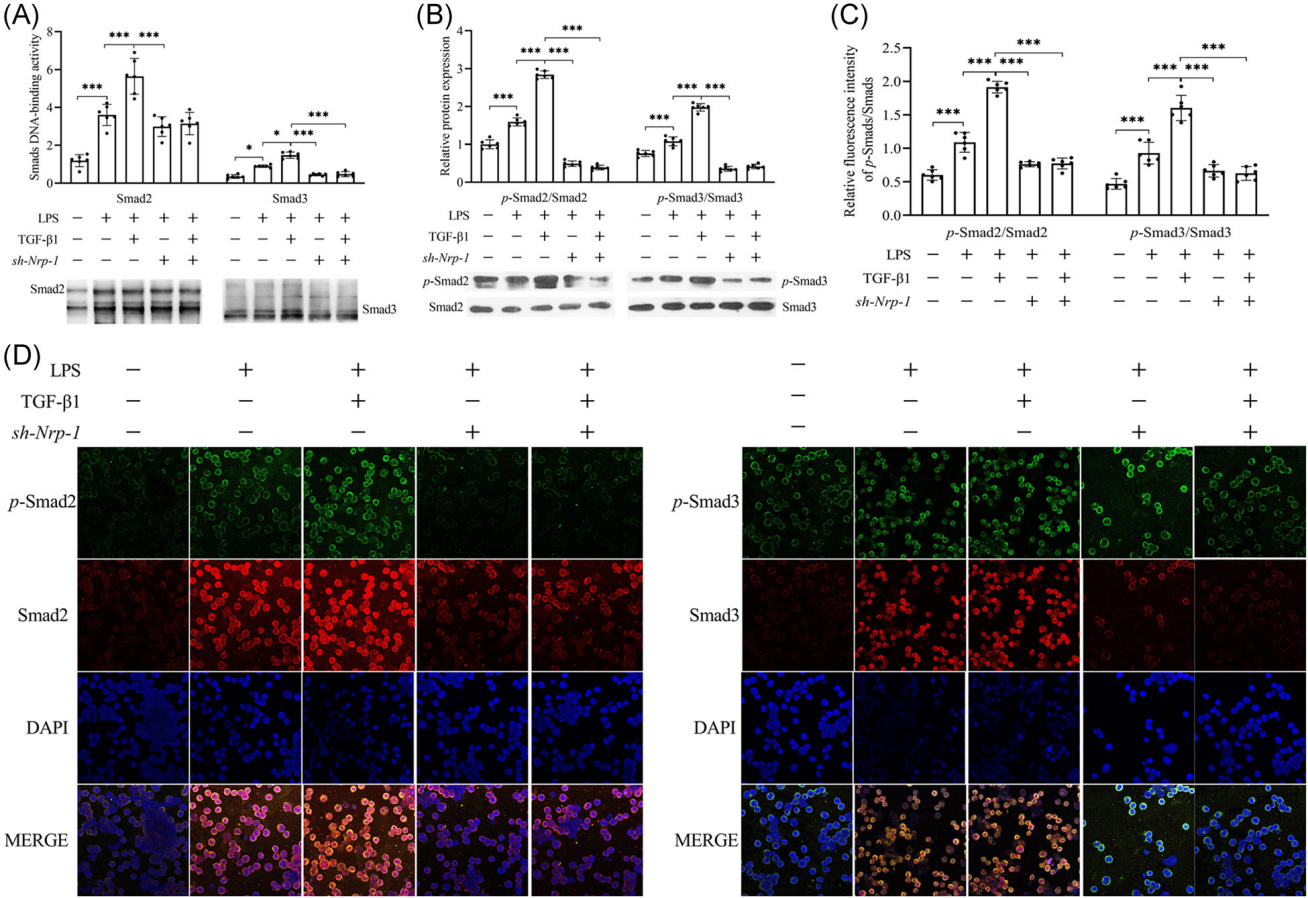
The effect of sh‐Nrp‐1 on the activity of TGF‐β1/Smads signal transduction pathway of CD4^+^CD25^+^ T_regs_ in sepsis induced by LPS. Recombinant TGF‐β1 induction enhanced the DNA‐binding activity of Smad2/3, the p‐Smad2/Smad2, and p‐Smad3/Smad3 ratios. Knockdown of Nrp‐1 with sh‐Nrp‐1 transfection in CD4^+^CD25^+^ T_regs_ of the spleen cells suppressed the recombinant TGF‐β1‐mediated the activity of the TGF‐β1/Smads signaling pathway. EMSA images and statistical figures of the DNA binding activity of Smad2/3 (A). Statistical figures and images that represent the western blot of p‐Smad2/Smad2 and p‐Smad3/Smad3 ratios (B). Representative immunofluorescence images and statistical figures of p‐Smad2/Smad2 and p‐Smad3/Smad3 (C, D)

The results of EMSA (Figure 3A) showed that the DNA‐binding capacity of Smad2/3 was stimulated due to recombinant TGF‐β1 as compared to the activity in the LPS administration alone group, especially Smad2 (*p* < .001). Western blot analysis (Figure 3B) and immunofluorescence analysis (Figure 3C,D) showed that the ratios of p‐Smad2/Smad2 and p‐Smad3/Smad3 were elevated greatly in CD4^+^CD25^+^ T_regs_ that were cultured in LPS for 48 h in comparison to the ratios noted in the control group (*p* < .001), while these phosphorylation ratios further rose upon the induction of recombinant TGF‐β1 in comparison to the LPS only treatment (*p* < .001). Compared to the LPS group, knockdown of Nrp‐1 resulted in a considerable reduction in the binding capability of DNA in Smad2/3 (*p* < .01 or 0.001), the p‐Smad2/Smad2 (*p* < .01 or 0.001) and p‐Smad3/Smad3 (*p* < .01 or 0.001) ratios. Compared with the only administration of recombinant TGF‐β1 group, Nrp‐1 silencing caused significant suppression of the recombinant TGF‐β1‐induced activity of the TGF‐β1/Smads signal transduction pathway of CD4^+^CD25^+^ regulatory T cells when recombinant TGF‐β1 and sh‐Nrp‐1 were co‐administered (*p* < .001).

### 3.4 Smad3 is the key contributing factor in the recombinant TGF‐β1‐mediated stability enhancement of CD4^+^CD25^+^ T_regs_ in the presence of LPS

**Figure 4:**
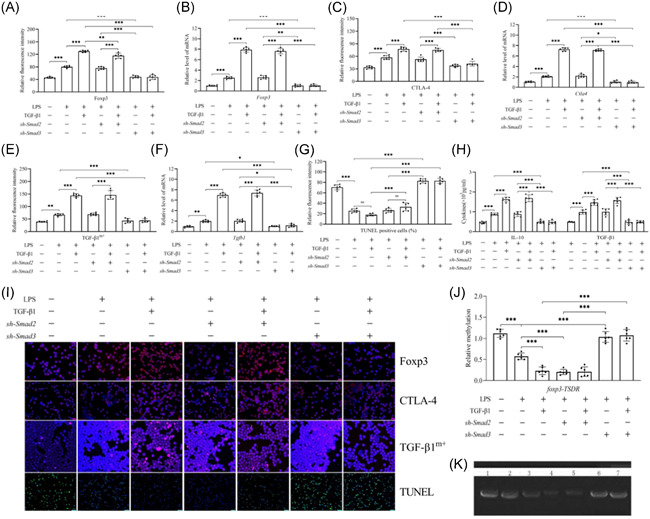
The effect of sh‐Smad2 or sh‐Smad3 transfection on recombinant TGF‐β1‐mediated increased stability of CD4^+^CD25^+^ T_regs_ in sepsis caused by LPS. The weakened stability of CD4^+^CD25^+^ T_regs_ after transfection with sh‐Smad3, but not sh‐Smad2. Transfection with sh‐Smad3, but not sh‐Smad2, using lentiviral infection destabilized the CD4^+^CD25^+^ T_regs_ upon LPS stimulation with recombinant TGF‐β1. Statistical figures of Foxp3 (A, B), CTLA‐4 (C, D), and TGF‐β1^m+^ (E, F) expressions at the gene and protein levels, and the level of CD4^+^CD25^+^ T_regs_ apoptosis (G). The TGF‐β1 and IL‐10 secretion levels (H). Representative immunofluorescence images of Foxp3, CTLA‐4, and TGF‐β1^m+^ expressions, along with the apoptotic level of CD4^+^CD25^+^ T_regs_ (I). The methylation of foxp3‐TSDR (J, K)

In comparison to the LPS group, CD4^+^CD25^+^ T_regs_ stability was weakened greatly after transfection with sh‐Smad3. This phenomenon included decreased expression of Foxp3 (Figure 4A,B,I), CTLA‐4 (Figure 4C,D,I), and TGF‐β1^m+^ (Figure 4E,F,I) at the gene and protein levels (*p* < .05 or 0.001), reduced levels of the secretion of TGF‐β1 and IL‐10 (Figure 4H, *p* < .001), increased apoptosis (Figure 4G,I, *p* < .001), as well as increased foxp3‐TSDR methylation (Figure 4J,K, *p* < .001). Smad2 knockdown by transfection with sh‐Smad2 in the CD4^+^CD25^+^ T_regs_ did not affect the stability of CD4^+^CD25^+^ T_regs_, hence, the LPS and sh‐Smad2 transfection groups were similar in this case (*p* > .05). Furthermore, compared with the only administration of recombinant TGF‐β1 group, silencing of Smad3, but not Smad2, suppressed significantly the stability of CD4^+^CD25^+^ T_regs_ that was stimulated by recombinant TGF‐β1 upon the coadministration of recombinant TGF‐β1 and sh‐Smad3 (*p* < .001).

### 3.5 Nrp‐1 is the key receptor contributing to the stimulated CD4^+^CD25^+^ T_regs_ stability caused by recombinant TGF‐β1/Smad3 signaling pathway in the presence of LPS

**Figure 5:**
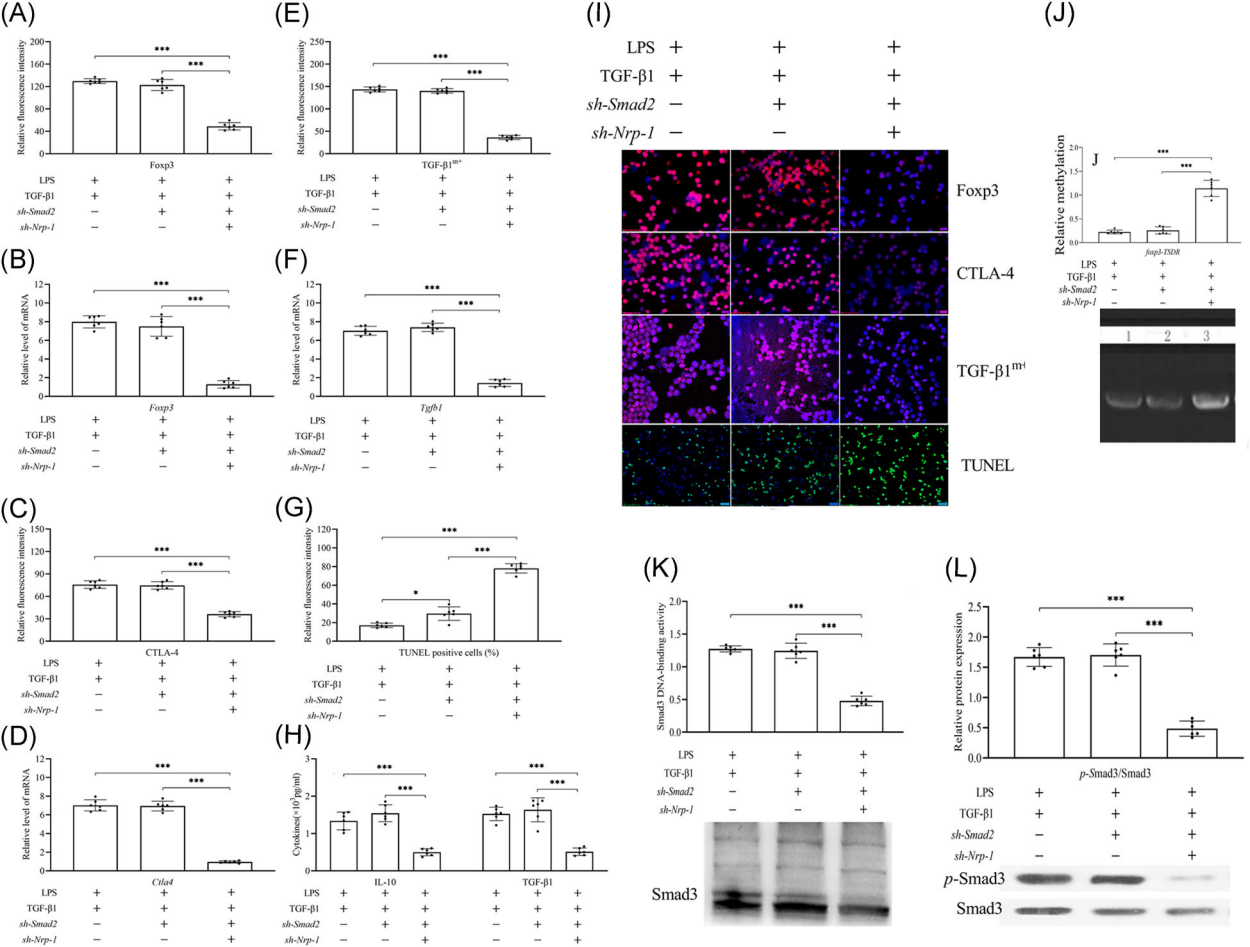
The effect of sh‐Nrp‐1 and sh‐Smad2 transfection on recombinant TGF‐β1‐mediated promotion of the stability and Smad3 of CD4^+^CD25^+^ T_regs_ in LPS‐induced sepsis. Smad2 knockdown did not affect recombinant TGF‐β1‐mediated stability, the DNA‐binding capacity Smad3, and ratios of p‐Smad3/Smad3 in CD4^+^CD25^+^ T_regs_. Nrp‐1 knockdown lowered the elevated stability levels of CD4^+^CD25^+^ T_regs_ caused by recombinant TGF‐β1 in the presence of sh‐Smad2 during LPS‐induced sepsis. Statistical figures of Foxp3 (A, B), CTLA‐4 (C, D), and TGF‐β1^m+^ (E, F) expressions at the gene and protein levels, and the apoptotic levels of CD4^+^CD25^+^ T_regs_ (G). The secretion levels of TGF‐β1 and IL‐10 (H). Representative immunofluorescence images of Foxp3, CTLA‐4, and TGF‐β1^m+^ expressions, along with the apoptotic level of CD4^+^CD25^+^ T_regs_ (I). The methylation of foxp3‐TSDR (J). Representative EMSA images and statistical figures of the DNA binding activity of Smad3 (K). Western blot images and statistical figures representing the p‐Smad3/Smad3 ratios (L)

Compared with coadministration of recombinant TGF‐β1 and sh‐Smad2 group, Nrp‐1 silencing weakened the stimulated levels of CD4^+^CD25^+^ T_regs_ stability that was caused by the recombinant TGF‐β1/Smad3 signaling pathway as compared to the results of coadministration of recombinant TGF‐β1, sh‐Smad2, and sh‐Nrp‐1 (Figure 5A–J, *p* < .001). EMSA (Figure 5K) and Western blot analysis (Figure 5L) results showed that knockdown of Smad2 could not affect recombinant TGF‐β1‐mediated DNA‐binding capacity of Smad3 and p‐Smad3/Smad3 ratios, there was no difference between recombinant TGF‐β1 with or without sh‐Smad2 transfection groups (*p* > .05). In comparison to the induction of recombinant TGF‐β1and coadministration of recombinant TGF‐β1 and sh‐Smad2 group, knockdown of Nrp‐1 caused notable weakening of the DNA‐binding activity of Smad3 (*p* < .001) and p‐Smad3/Smad3 (*p* < .001) ratios.

## 4 DISCUSSION

Sepsis can induce an immunosuppressive state,^1^, ^2^, ^3^ which can further progress to septic shock and MODS.^1^ Increasing evidence has indicated that changes in CD4^+^CD25^+^ T_regs_ numbers and function lead to immune disorders.^5^, ^14^, ^15^, ^16^ Nrp‐1 promotes the stability of T_regs_ through an Nrp‐1/Sema3A axis.^10^, ^17^ T_regs_ mediate immunosuppression and enhance the susceptibility to secondary infections. Studies have found that accumulation of T_regs_ is associated with TGF‐β1, and treatment with TGF‐β1 inhibitors results in reduced concentrations of T_regs_.^18^, ^19^ Nrp‐1 is a high‐affinity TGF‐β1 receptor and mediates T_regs_‐regulated immunosuppression.^11^, ^12^, ^13^ However, the potential mechanism and signaling pathways underlying the stabilizing effects of Nrp‐1 on T_regs_ in immunosuppression remain unclear. Here, we report that Nrp‐1 and TGF‐β1 stimulate the stability of CD4^+^CD25^+^ T_regs_ in the presence of LPS and that Nrp‐1 contributes to the activity of recombinant TGF‐β1 by the regulation of TGF‐β1/Smads cell signaling in sepsis.

TGF‐β1 is well‐documented as an anti‐inflammatory cytokine with important roles in various inflammatory processes.^18^, ^19^ Experiments with neutralizing antibodies have shown that blocking TGF‐β1^m+^ inhibits immunosuppression. Liu et al.^20^ found that the frequency of Foxp3^+^ thymocytes was markedly reduced in a mouse model lacking the TGF‐β1^m+^ type I receptor (TbRI), while Shen demonstrated that treatment with a TGF‐β1^m+^‐specific inhibitor caused a percentage decrease of T_regs_ in liver tissue.^21^ The present research verified that TGF‐β1 induction promoted T_regs_ stability, which included a decrease in the apoptotic rate, increased viability, and increased expression of T_regs_ biomarkers. Ctla4 KO mice and Foxp3‐deficient scurfy mice display a similar pathology and develop the same autoimmune‐like syndrome, indicative of the importance of the role played by CTLA‐4 and Foxp3 in immunosuppression.^22^, ^23^ The expression of Foxp3 distinguishes T_regs_ from other immune cells.^10^ Foxp3 stabilizes the suppressive phenotype and capabilities of T_regs_. Foxp3 and CTLA‐4 can serve as markers to characterize CD4^+^CD25^+^ T_regs_. IL‐10 functions both upstream and downstream of TGF‐β1 signaling. For instance, TGF‐β1 expression and secretion in T cells of the lamina propria can be induced by IL‐10. Additionally, IL‐10 and TGF‐β1 work together to mediate T_regs_ differentiation, which subsequently produces more of these, and maintaining the immune homeostasis of T_regs_.^24^, ^25^ This study reports an increase in the expression levels of Foxp3/CTLA‐4/TGF‐β1^m+^ in CD4^+^CD25^+^ T_regs_, as well as the secretion levels of TGF‐β1 and IL‐10 in the presence of LPS. The stability and negative immunosuppression function of T_regs_ and Foxp3 expression depends on the foxp3‐ TSDR's methylation status.^26^ This region in T_regs_ is more stable with elevated demethylation but in all other blood cells foxp3‐ TSDR is heavily methylated.^26^, ^27^ Natural and most of the induced T_regs_ showed stability and a demethylated foxp3‐TSDR region, and both the populations of T_regs_ displayed weakened functions in healthy mice and mice suffering from sepsis.^15^ This study also reports a significantly reduced methylation level of foxp3‐TSDR in CD4^+^CD25^+^ T_regs_ in the presence of LPS or in combination with recombinant TGF‐β and stimulated demethylation of foxp3‐TSDR.

The Nrp‐1 expression in T_regs_ is associated with T_regs_‐mediated immunosuppression.^15^, ^17^ Some studies have revealed the involvement of Nrp‐1 in the immune system response and the regulation of the immunological synapse.^10^, ^28^ Nrp‐1 is a key factor in controlling cerebral angiogenesis, cancer generation and recurrence, and fibrosis of the liver by regulating the TGF‐β1 pathway.^11^, ^12^, ^13^, ^29^, ^30^ Nrp‐1 antagonists have immunoregulatory effects through reducing TGF‐β1 production in T_regs_.^14^, ^15^ This study demonstrates that Nrp‐1 knockdown lessens the viability of CD4^+^CD25^+^ T_regs_ during sepsis depending upon its grade and time. When CD4^+^CD25^+^ T_regs_ were treated with recombinant TGF‐β1, the viability of CD4^+^CD25^+^ T_regs_ was markedly enhanced. After transfection with various concentrations of sh‐Nrp‐1, the viability of CD4^+^CD25^+^ T_regs_ got reduced. Results indicated that knockdown of Nrp‐1 reduced the expression levels of Foxp3/CTLA‐4/TGF‐β1^m+^ and secretion levels of IL‐10 and TGF‐β1, and increased apoptotic rates, along with methylation rate of foxp3‐TSDR, in CD4^+^CD25^+^ T_regs_. The stability of CD4^+^CD25^+^ T_regs_ is mediated by the stable expression of Foxp3 and negative immunoregulation. Therefore, our results indicate that Nrp‐1 and TGF‐β1 significantly contribute to the stability of CD4^+^CD25^+^ T_regs_.

Increasing evidence has indicated that the TGF‐β1/Smads signaling pathway is activated during infection, especially via Smad2/3.^11^, ^12^, ^13^ Data have suggested that the T_regs_/Th17 balance may be regulated by the TGF‐β/Smads signaling pathway.^31^ In this study, we showed that the application of recombinant TGF‐β1 improved the binding ability of the DNA of Smad2/3, along with p‐Smad 2/Smad 2 and p‐Smad 3/Smad 3 ratios, whereas knockdown of Nrp‐1 yielded the opposite effect. Smad3 is a key factor in maintaining the stability of T_regs_ and exerting negative immunomodulatory function. Using a model system for analyzing Foxp3 induction, Tone et al.^32^ demonstrated that the transcription factor Smad3 was required for Foxp3 enhancer activity. This also explains the effect of TGF‐β1 on Foxp3^+^T_regs_ function. Xiao et al.^33^ also observed that all‐trans retinoic acid (ATRA) enhanced Smad3 expression and phosphorylation that resulted in increased differentiation of T_regs_. In this study, we demonstrated that Smad3 was the key factor in stabilizing CD4^+^CD25^+^ T_regs_ mediated by recombinant TGF‐β1 during sepsis. The CD4^+^CD25^+^ T_regs_ stability weakened after transfection with sh‐Smad3. Furthermore, Smad3 silencing decreased the stability of CD4^+^CD25^+^ T_regs_ that was increased by the TGF‐β1.

In conclusion, we demonstrated the cooperative exertion of Nrp‐1 and TGF‐β1 regulatory impact on the stability of CD4^+^CD25^+^ T_regs_ via enhancing the Smad2/3 activity and phosphorylation in the presence of LPS. Nrp‐1 is beneficial for TGF‐β1 to enhance the stability of regulatory CD4^+^CD25^+^ T cells and may represent a novel therapeutic target with the potential to improve the CD4^+^CD25^+^ T_regs_‐related primary negative immunoregulation associated with the TGF‐β1/Smads signaling pathway, especially Smad3, in sepsis.

## CONFLICT OF INTERESTS

The authors declare that there are no conflict of interests.

## AUTHOR CONTRIBUTIONS

Yulei Gao and Yanfen Chai: Funding acquisition. Yulei Gao, Xiang Zhang, and Yanfen Chai: Planned the study and Wrote the protocol. Yulei Gao, Xiang Zhang, Ziyi Wang, and Yanfen Chai: Collected the data, Performed statistical analyses, and Contributed to writing the manuscript. Ziyi Wang and Yuting Qiu: Did technical work. Yancun Liu and Songtao Shou: Helped with data collection, study design, and coordinated the study. All authors read and approved the final manuscript.

## Data Availability

The data that support the findings of this study are available from the corresponding author upon reasonable request.
